# Deciphering of Pod Borer [*Helicoverpa* *armigera* (Hübner)] Resistance in *Cajanus* *platycarpus* (Benth.) Offers Novel Insights on the Reprogramming and Role of Flavonoid Biosynthesis Pathway

**DOI:** 10.3390/toxins14070455

**Published:** 2022-07-02

**Authors:** Shaily Tyagi, Maniraj Rathinam, Pathour Rajendra Shashank, Nidhee Chaudhary, Ajit Kumar Shasany, Rohini Sreevathsa

**Affiliations:** 1 ICAR-National Institute for Plant Biotechnology, Pusa Campus, New Delhi 110012, India; shailys2709@gmail.com (S.T.); rmani607@gmail.com (M.R.); akshasany@gmail.com (A.K.S.); 2 Centre for Biotechnology and Biochemical Engineering, Amity Institute of Biotechnology, Amity University, Noida 201313, India; nchaudhary@amity.edu; 3 Division of Entomology, ICAR-Indian Agricultural Research Institute, Pusa Campus, New Delhi 110012, India; spathour@gmail.com

**Keywords:** anthocyanins, pigeonpea, flavonoids, herbivory, wild relatives

## Abstract

Management of pod borer, *Helicoverpa armigera* in pigeonpea (*Cajanus cajan* L.), an important legume crop, has been a pertinent endeavor globally. As with other crops, wild relatives of pigeonpea are bestowed with various resistance traits that include the ability to deter the *H. armigera.* Understanding the molecular basis of pod borer resistance could provide useful leads for the management of this notorious herbivore. Earlier studies by our group in deciphering the resistance response to herbivory through multiomics approaches in the pigeonpea wild relative, *Cajanus platycarpus,* divulged the involvement of the flavonoid biosynthesis pathway, speculating an active chemical response of the wild relative to herbivory. The present study is a deeper understanding of the chemical basis of pod borer (*H. armigera*) resistance in, *C. platycarpus,* with focus on the flavonoid biosynthesis pathway. To substantiate, quantification of transcripts in *H. armigera*-challenged *C. platycarpus* (8 h, 24 h, 48 h, 96 h) showed dynamic upregulation (up to 11-fold) of pivotal pathway genes such as chalcone synthase, dihydroflavonol-4-reductase, flavonoid-3′5′-hydroxylase, flavonol synthase, leucoanthocyanidin reductase, and anthocyanidin synthase. Targeted LC-MS analyses demonstrated a concomitant increase (up to 4-fold) in naringenin, kaempferol, quercetin, delphinidin, cyanidin, epigallocatechin, and epicatechin-3-gallate. Interestingly, *H. armigera* diet overlaid with the over-produced flavonoids (100 ppm) showed deleterious effects on growth leading to a prolonged larval period demonstrating noteworthy coherence between over-accumulation of pathway transcripts/metabolites. The study depicts novel evidence for the directed metabolic reprogramming of the flavonoid biosynthesis pathway in the wild relative to pod borer; plant metabolic potential is worth exploiting for pest management.

## 1. Introduction

Pigeonpea (*C. cajan* (L.) Millisp.), is one of the major legume crops belonging to the family Fabaceae and is sown in the semi-arid tropics [1]. Pigeonpea seeds are a major source of proteins for the African and Asian population. Globally, India is the major producer of pigeonpea which accounts for 70% of production followed by Myanmar, Malawi, Kenya, and Tanzania. Besides its economic and medicinal significance, the crop can grow in vast climatic conditions all over the year. The crop has been highly profitable to farmers due to its growing capacity, adaptability, and decreased cultivation costs. Owing to this, the demand for pigeonpea has augmented with the increase in population [2,3]. Despite its amplified cultivation, stagnated productivity due to biotic and abiotic stresses has been a continued concern. One of the chief threatening factors for the stagnated productivity in pigeonpea has been the pod borer, *H. armigera* which causes serious economic losses [1].

To meet the demand and bridge the yield gap, realization of the potential yield and mitigation of pests as notorious as the polyphagous *H. armigera* is the need of the hour. Considering transgenic technology as one of the options for stress mitigation and in lieu of the concerns pertaining to the technology, alternate strategies are required to be in place. Hence, the use of biological molecules as bio pesticides and/or the proteins producing these can form lucrative options for pest management. In this regard, interpreting robust chemical-based pod borer resistance mechanisms that are ought to keep the insect at bay assumes importance.

Crop wild relatives are known to harbor agronomically important traits [4,5] which form an efficient and ecofriendly approach for pest management. The genus *Cajanus* consists of a single cultivated *C. cajan* (pigeonpea) with the rest being wild relatives in different gene pools [6,7]. *C. platycarpus* is a wild relative of pigeonpea from the tertiary gene pool that possesses a plethora of resistance traits [8,9,10], including towards the pod borer [11]. While trying to decipher the mechanism of pod borer resistance in this species through a multiomics approach, we stumbled upon a tightly regulated phenylpropanoid pathway that was linked with the antibiosis in the pod borer [12]. This implicated the wild relative to have evolved an insect-specific chemical defense to encounter the herbivore.

The phenylpropanoid metabolic pathway produces >8000 metabolites involved in plant development, as well as in environmental interactions [13]. The pathway branches out into the production of lignins and flavonoid biosynthesis that produces secondary metabolites such as flavanones, flavonols, flavanols, and anthocyanins. It has also been established that the pathway undergoes modulation due to various factors including environmental stresses [14]. Normally, the chief components of the chemical barrier constituted by plants to fight herbivory include flavonoids, which are the secondary metabolites that are well connected to plant defense systems [15,16]. These metabolites often are anti-digestive, anti-feedants, and can also lead to toxic effects on the insect’s feeding, growth, development, and reproduction [17,18].

It would hence be worthwhile to understand the pattern of genes/metabolites of the flavonoid pathway reinforcing the chemical basis of pod borer resistance in *C. platycarpus.* The present study was therefore envisaged to assess holistically, the correlation in the expression of pivotal herbivore-responsive genes of the flavonoid pathway as well as the accumulation of concomitant metabolites during the continued herbivory on *C. platycarpus*. Furthermore, the question was to assess any further link to their dynamic accumulation and their effect on *H. armigera.* Such an understanding of intricate plant biology is necessary prior to metabolic/genomic engineering for pest resistance. The analyses revealed that the expression of genes in the flavonoid pathway were tightly linked to the dynamic accumulation of the metabolites during the continued herbivory in *C. platycarpus.* Information emanating from this study can be strategically incorporated into crop improvement programs and used to engineer pod borer resistance in the cultivated pigeonpea.

## 2. Materials and Methods

### Plant Material and Herbivore Challenge by H. armigera

Seeds of the pigeonpea wild relative, *C. platycarpus* (ICPW 068) were procured from ICRISAT, Hyderabad, India, and used for experimental work in the present study. Seeds were sown in plastic pots (35 cm diameter × 40 cm height) containing approximately 15 kg of soil (3:1 soil and manure mix). The pots were maintained at 100% field capacity (FC) along with 24% water holding capacity. The experimental plants were maintained under greenhouse conditions and ensured that the plants were not stressed prior to herbivory.

*H. armigera* larvae collected from pigeonpea growing fields of the institute were maintained in the laboratory. The insects were reared on an artificial diet [19] and maintained under controlled conditions of 25 ± 5 °C, 70 ± 10% RH, and 16 h/8 h day and light photoperiod. The adults were retained in glass jars (50 × 50 cm) with paper sheets for oviposition and fed with honey (10%) embedded in cotton and placed in Petri dishes at the bottom of the jars. *H. armigera* egg masses were collected and further used for maintaining the culture as well as for experimental purposes.

Forty-five days old plants of *C. platycarpus* were challenged with five 2nd instar *H. armigera* larvae. The larvae were released onto the leaves of plants that were covered with a transparent plastic sheet to avoid larval escape [20]. Based on earlier studies [21], leaves of challenged plants were collected at different time intervals (8 h, 24 h, 48 h, 96 h) along with control leaves which were collected prior to challenging (0 h). Four biological replicates were maintained and challenged for each time point. The collected leaves were frozen in liquid nitrogen and preserved at −80 °C until further studies.

## 3. Expression Analysis

### 3.1. Identification of Flavonoid Pathway Genes from C. platycarpus Transcriptome Data

Transcripts pertaining to flavonoid metabolism were selected based on the BLAST annotation of in-house-generated *C. platycarpus* transcriptome data [21]. The 8 selected flavonoid metabolism genes were chalcone isomerase (CHI–4 isoforms), chalcone synthase (CHS–3 isoforms), dihydroflavonol 4-reductase (DFR–7 isoforms), flavonoid 3′5′-hydroxylase (F3′5′H–2 isoforms), flavonol synthase (FLS–3 isoforms), leucoanthocyanidin reductase (LAR–2 isoforms), anthocyanidin synthase (LDOX/ANS–2 isoforms) and UDP flavonoid glycosyltransferase (UFGT–4 isoforms). Further, the selected transcripts were cross-checked with the pigeonpea genome, and the presence of possible domains was confirmed with NCBI CD (conserved domain) database.

### 3.2. Total RNA Isolation and cDNA Synthesis

Approximately 100 mg of leaf tissue was crushed in liquid nitrogen to a fine powder using a mortar and pestle. Total RNA was extracted from different samples by Spectrum ^TM^ total RNA isolation kit (Sigma-Aldrich, St. Louis, MO, USA) as per the manufacturer’s instructions. The DNA contamination was removed by on-column DNase I (Sigma-Aldrich, St. Louis, MO, USA) treatment. The extracted RNA was quantified using NanoDrop 2000 spectrophotometer (Wilmington, DE, USA) and verified by 0.8% agarose gel electrophoresis for quantity, quality as well as purity of RNA. cDNA synthesis was carried out using 2.5 µg of total RNA following the manufacturer’s instructions (SuperScript^®^ VILO^TM^; Invitrogen, Carlsbad, CA, USA).

### 3.3. Real-Time PCR

The selected 8 genes and their respective isoforms identified from the in-house generated transcriptome data of *C. platycapus* under continued herbivory were used for expression analysis using qRT-PCR (AriaMx Real-Time PCR system; Agilent, Santa Clara, CA, USA). Different gene-specific primers (Table S1) along with the reference gene, *Initiation factor 4α* (*IF4α*) [22] were used for the reaction. The qRT-PCR conditions were as follows: initial denaturation at 95 °C for 5 min, followed by 40 cycles each of 95 °C for 10 s, 15 s at 60 °C and 15 s at 72 °C. Four independent biological and two technical replicates along with a non-template control were used in this study. Data analysis was executed by considering 0 h as the baseline along with 8 h, 24 h, 48 h, and 96 h as test timepoints. The internal reference gene was used for data normalization followed by fold change calculation [23].

## 4. Copy Number Assessment of the Selected Flavonoid Biosynthesis Genes in *C. platycarpus*

### 4.1. Probe Designing

Complete coding sequences of selected genes were subjected to BLAST alignment with the available pigeonpea genome using NCBI genome BLAST. The longest exon region was selected for primer design (Table S2). The desired gene fragments were amplified from 100 ng cDNA from *C. platycarpus*. The eluted and purified PCR products were further used for the Digoxigenin (DIG)-labeled probe synthesis and as positive controls in genomic Southern analysis.

### 4.2. Genomic Southern Analysis

To identify the gene copy number of the flavonoid biosynthesis pathway genes in *C. platycarpus*, genomic DNA from young and tender leaves was isolated following the CTAB method [24]. For each gene, around 15 μg high-quality DNA was separately digested overnight with any two restriction enzymes (*Hind*III, *Kpn*I, and *Bam*HI; New England Biolabs, Ipswich, MA, USA). The restricted DNA samples were separated by gel electrophoresis using 0.8% agarose gel in TAE buffer (Tris-acetate EDTA; Millipore Sigma, Burlington, MA, USA). The electrophoretically separated DNA fragments were further transferred onto a positively charged nylon membrane (Millipore Sigma, Burlington, MA, USA) by capillary action for 18 h in 20X SSC. The respective dig-labeled probes were used for hybridization. Hybridization, washing, blocking, antibody binding, and detection were carried out according to the manufacturer’s instructions (Roche Holding AG, Basel, CH).

### 4.3. Targeted Quantitative Estimation of Flavonoids and Phenylpropanoids in C. platycarpus during H. armigera Infestation

The frozen leaf tissues of challenged plants (0 h, 8 h, 24 h, 48 h, 96 h) were used to study altered metabolites during the insect invasion. Flavonoids and phenylpropanoids were extracted with 80% methanol overnight at room temperature under brief agitation. The resulting extracts were subjected to centrifugation at 6010 rcf for 10 min at 4 °C; supernatants were transferred to a fresh reaction tube and then vacuum-dried at 65 °C in a SpeedVac. The dried form of extracts was later resuspended in 80% methanol. Analyses of target flavonoids (quercetin, myricetin, kaempferol, delphinidin, pelargonidin, naringenin, cyanidin, malvidin, rutin, catechin, epicatechin-3-gallate, and epigallocatechin) and phenylpropanoids (cinnamic acid, t-ferulic acid, p-coumaric acid, and caffeic acid) were performed as described in previous studies [25]. Methanolic extracts were used for the quantification of individual flavonoids. LC-MS analysis of samples was carried out in a UPLC system (Exion LC, Sciex, Framingham, MA, USA) coupled to a triple quadrupole system (QTRAP6500+; ABSciex, Framingham, MA, USA) using electrospray ionization. For positive ionization, the voltage was set at 5500 V. The values of gas 1 and gas 2 (70 psi), curtain gas (40 psi), collision-assisted dissociation (medium), and temperature of the source (650 °C) were used. The mass spectrometer was used in multiple reaction monitoring modes (MRM) for qualitative and quantitative analysis using analytical standards of flavonoids (Merck, Hunterdon County, NJ, USA). Analyst software (version 1.5.2) (Sciex, Framingham, MA, USA) was used for the identification and quantitative analysis. Each targeted metabolite was profiled in 3 biological replicates.

### 4.4. Assessment of the Biological Activities of Selected Flavonoids on the Growth and Development of H. armigera

The effect of flavonoids on the growth and development of *H. armigera* was studied by feeding the 2nd instar larvae (7 days old) on a flavonoid-incorporated artificial diet. Seven flavonoids: delphinidin, pelargonidin, naringenin, cyanidin, malvidin, epicatechin-3-gallate, and epigallocatechin (Sigma-Aldrich, St. Louis, MO, USA) were assessed using diet incorporation assay [26] with slight modifications. Based on the solubility, epicatechin-3-gallate and epigallocatechin were dissolved in water; cyanidin and malvidin in 1% DMSO; naringenin in 1.5% DMSO; delphinidin, pelargonidin were dissolved in 2% DMSO. The different flavonoids were weighed, dissolved, and mixed with the diet just after its preparation. Larvae of *H. armigera* (~0.5 cm in length) were released on the diet containing two concentrations of each flavonoid (10 and 100 ppm). These doses of flavonoids are within the physiological range (20–1600 ppm) of their natural production from various plant sources [27]. A single larva was released in an individual 30 mm plastic Petri plate. Ten replications were maintained for each treatment with ten (seven days old) individual larvae in each replication. Larvae fed on an untreated diet were maintained as a control. Eight days after treatment (DAT), larval weight, length, and duration of the larval stage were recorded. The whole experiment was maintained at a constant temperature of 25 ± 5°C with a relative humidity of 70 ± 10% and 16 h/8 h light and dark photoperiod 4.5 Statistical analyses.

All statistical analyses were performed in Microsoft Excel using the “data analysis” package. For all analyses, the standard deviation between the biological replicates were calculated and error bars were made. The student *t*-test was performed to determine significant (*p <* 0.05, *p* < 0.001) difference between time points.

## 5. Results and Discussion

Plants being sessile are exposed to a multitude of abiotic and biotic stresses during their growth period. In response, plants have devised highly strategic and intricate molecular responses [12,28,29,30] that result either in stress mitigation or death of plants. Insect pests have always been a menace in agriculture as their infestation results in enormous crop losses globally. In this regard, host plant resistance involving both morphological and biochemical traits has been found to be effective at minimizing damage from pests such as the notorious *H. armigera* [21,31,32,33].

Herbivore-associated molecular patterns (HAMPs) activate the early signaling events in plants leading to specific responses determined by physical barriers, secondary metabolites, or defense proteins that interact with feeding, digestion, and development in insects. In the recent past, the development of genomics and molecular biology tools has instigated scientists to unravel the molecular mechanisms supporting these interactions.

**Figure 1:**
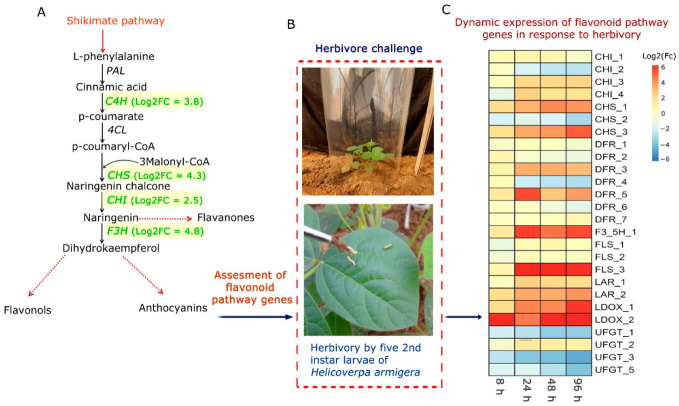
Dynamic response of flavonoid biosynthesis pathway genes in *C. platycarpus* under continued herbivory: (**A**) Pathway map depicting key genes (green color) upregulated in *C. platycarpus* compared to *Cajanus cajan* assessed earlier in the comparative proteome profiling during herbivory (obtained from proteome data; Rathinam et al., 2020 [33]. C4H: Cinnamate-4-hydroxylase, CHS: Chalcone synthase, CHI: Chalcone flavanone isomerase, F3H: Flavanone 3-hydroxylase involved in the anti-herbivore response of *C. platycarpus*; Log2FC in parenthesis indicates the upregulation of the respective genes in the wild relative based on differential proteomic analysis *vis a vis* cultivated pigeonpea (**B**) Experimental setup used to understand the dynamic changes in flavonoid pathway genes and metabolites under continued herbivory in the wild relative; (**C**) qRT-PCR analyses of the herbivore- challenged samples depicting the response of flavonoid biosynthesis genes in *C. platycarpus*.

In order to maintain nutritional quality as well as crop productivity, various improvement programs have been adopted. In this regard, crop wild relatives have gained importance due to them harboring useful traits and are projected as potential candidates for the development of insect-resistant plants [34,35]. In our laboratory, we have been making focused efforts to decipher the molecular mechanism underlying pod borer resistance in one of the pigeonpea wild relatives, *C. platycarpus* using multiomics approaches [21,33,36]. While studying the dynamic transcriptome and proteome data during continued herbivory, we observed that a strong chemical-based resistance response prevailed in the wild relative compared to the cultivated counterpart. This was because, skewed upregulation of the phenylpropanoid (PP)-flavonoid biosynthesis pathway genes were found in the wild relative on a dynamic basis (Figure 1A). Differential proteomic analysis of the wild relative *vis a vis* the cultivated pigeonpea demonstrated a 2–5-fold increase in the production of pivotal proteins in the phenylpropanoid pathway (Figure 1A) [33]. This prompted us to dig deeper into the involvement of the PP-flavonoid pathway in the resistance response and understand their dynamics at the transcriptome and metabolite level (Figure 1B), as this would specifically divulge clues about the intricate metabolic reprogramming if present in the resistance response of the wild relative. Since our earlier findings depicted differential upregulation of the flavonoid pathway genes in the wild relative *vis a vis* cultivated pigeonpea, the present study only focused on the wild relative.

## 6. Dynamic Expression of Flavonoid Pathway Genes in *C. platycarpus* in Response to *H. armigera*

The focus of this study was therefore to assess the extent of involvement of flavonoid biosynthesis pathway genes of *C. platycarpus* in their response to *H. armigera*. In this regard, 45 days old plants of the wild relative were challenged with *H. armigera,* and leaves were sampled at four time points (8 h, 24 h, 48 h, 96 h). Based on our previous findings [21,33], a total of 8 genes and their isoforms as listed previously were selected from the transcriptome data to assess their dynamic expression. Since we had earlier assessed the dynamic response of *C4H* and *F3H* (Figure 1A) [33], in the comparative proteomic analysis of *C. platycarpus* and *C. cajan* during herbivory, we selected other pivotal genes of the pathway. qRT-PCR analysis demonstrated that higher expression (4–11-fold increase) was seen in CHS_1, CHS_3, DFR_3, DFR_5, F3′5′H_1, FLS_3, LAR_2, LDOX_1, LDOX_2 followed by moderate expression (1–2-fold increase) in CHI_3, CHI_4, LAR_1 and low/no change in expression (<1-fold increase) in CHI_1, CHI_2, CHS_2, DFR_1, DFR_2, DFR_4, DFR_6, DFR_7, FLS_1, FLS_2, UFGT_1, UFGT_2, UFGT_3, UFGT_5 (Figure 1C). Genes showing >4-fold up-regulation at any two time points were considered as significantly regulated. Based on the expression analyses, it was observed that isoforms of CHS, DFR, F3′5′H, FLS, and LDOX were upregulated to as high as 11-fold with continued herbivory (Figure 1C). Therefore, these genes were seemingly involved in the resistance response of the wild relative to herbivory. It was also interesting to find that specific isoforms of these genes were responding to herbivore infestation suggesting their participation in the resistance response. Several studies have demonstrated the involvement of flavonoid biosynthesis genes in the response to insect attacks [37,38,39,40] and have also established their role in the resistance response. However, a comprehension of their involvement in the host plant resistance to pod borer, especially in the legumes, has not yet been deciphered. Furthermore, it was important to assess whether the dynamic gene expression also extended to the accumulation of metabolites in response to herbivory. This information not only is a revelation of the molecular pattern of expression of pivotal genes during herbivory but also can form a basis for metabolic engineering for insect resistance in the cultivated pigeonpea.

## 7. Flavonoids and Phenylpropanoids Are Differentially Accumulated during the Dynamic Response of *C. platycarpus* to *H. armigera*

Metabolite profiling of plant tissues under various stresses has resulted in a better understanding of the plant-stress interactions. Plants are seen to produce a plethora of specialized metabolites, presumably to manage the attacking pests. Of the very many metabolites, are flavonoids which are a set of secondary metabolites with roles in core plant processes such as nodulation and attracting pollinators as well as in mitigation of various biotic and abiotic stresses [41,42,43].

**Figure 2:**
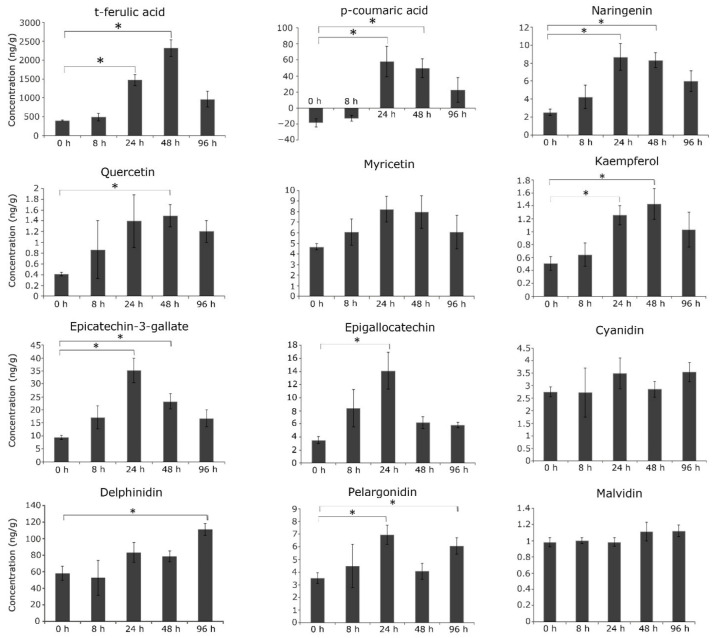
Targeted LC-MS profiling of phenylpropanoids and flavonoids under continued herbivory in *C. platycarpus*. The compounds were quantified by developing calibration and multiple reaction monitoring (MRM) of authentic standards. The graph shows values ±SD of three biological replicates from each sample. * depicts the significant difference between each time interval at *p* < 0.05, from the student’s *t*-test.

In order to assess the accumulation of secondary metabolites and their correlation with gene expression in the wild relative, we focused on the targeted profiling of flavonoids and phenylpropanoids in *C. platycarpus*. Among the targeted phenylpropanoids i.e., cinnamic acid, *t*-ferulic acid, *p*-coumaric acid, and caffeic acid, it was found that *t*-ferulic acid showed gradual and higher accumulation (5.7 times; *p <* 0.05) at 48 h compared to other metabolites (Figure 2). Another very interesting finding of our study demonstrated that while *p*-coumaric acid did not show any detectable accumulation at 0 h (without herbivory), the metabolite showed increased accumulation by 24 h (*p <* 0.05) *vis a vis* 48 and 96 h of continued herbivory. However, it was observed that cinnamic acid and caffeic acid were below the standard concentration and were hence not detected. This indicated that *t*-ferulic acid and *p*-coumaric acid could play an important role in the resistance response as they are important components that continue into the pathway for the production of secondary metabolites (Figure 2). The overproduction of metabolites from the general phenylpropanoid pathway that act as branch points to lignin/flavonoid pathways asserted the metabolic flux in the wild relative to the over-production of flavonoids.

**Figure 3:**
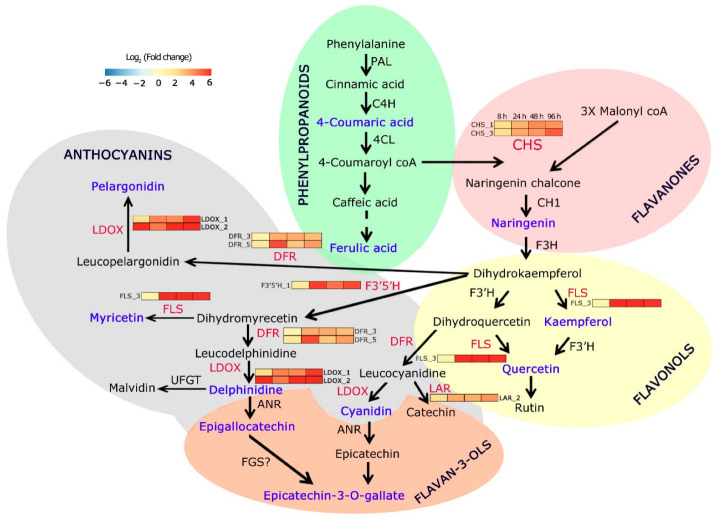
Pathway mapping of herbivore-induced flavonoid pathway genes and metabolites in *C. platycarpus*. Heat maps indicate the dynamic expression of respective genes at different time points (8 h, 24 h, 48 h, 96 h) of herbivory. The red-colored font denotes the genes assessed for expression analyses in the present study. The blue-colored font denotes over-accumulated metabolites during herbivory.

Further, the accumulation of various flavonoids varied with the time of herbivory (Figure 2). While some of the flavonoids showed maximum accumulation at earlier time points, some showed a progressive increase. Naringenin, myricetin, and pelargonidin showed higher accumulation (4, 2, 2 times respectively; *p <* 0.05) at 24 h of herbivory, whereas quercetin and kaempferol accumulated (2.5 and 2 times; *p* < 0.05) by 48 h (Figure 3). This implicated that these metabolites could be involved in early defense response to *H. armigera*. Additionally, epicatechin-3-gallate and epigallocatechin also showed higher accumulation (3.3 times; *p* < 0.05) at 24 h suggesting early response. However, a basal level of accumulation was seen with cyanidin and malvidin. Delphinidin showed higher accumulation (*p* < 0.05) at 96 h compared to other time points depicting its role in the continued herbivory. Irrespective of all the metabolites, rutin and catechin were constantly less accumulated or not detected.

It is known that, as a response to herbivory, plants initiate the de novo synthesis of defense compounds to deter the attacker. Independent studies have elaborated on the production and accumulation of flavonoids during various stresses [44,45,46]. However, our study delineated the specific pattern of metabolic reprogramming in the pigeonpea wild relative when the plant was under the continued attack by *H. armigera.* This emphasized the role played by flavonoids in the resistance response.

Our findings demonstrated that the expression of flavonoid pathway genes and accumulation of metabolites were tightly regulated in *C. platycarpus* during its encounter with *H. armigera*. Upon integration of both the gene expression as well as the metabolome data, an explicit correlation was seen in the upregulated expression of the genes and their corresponding metabolites establishing an agile and stereotypical response of the wild relative to the herbivore (Figure 3). Fascinatingly, pivotal branch point enzymes in the flavonoid biosynthesis pathway, initially CHS, a pertinent player in the production of flavonones such as naringenin and later flavonols such as quercetin and kaempferol was seen to be upregulated coordinating with the over-accumulation of the metabolites. The wild relative also depicted reallocation of resources for the overexpression of specific isoforms of F3′H, FLS, F3′5′H, and DFR that compete with dihydrokaempferol for the initiation of the production of anthocyanins (Figure 3) [47,48,49]. Fine orchestration of molecular events was seen in the pigeonpea wild relative with strategic channeling of resources for the significant (*p* < 0.05) accumulation of epicatechin-3-gallate and epigallocatechin to the maximum extent through the induced expression of LDOX and F3′5′H (Figure 3). Studies emerging from the literature have provided evidence for the role of these flavonoids in the mitigation of pathogens and insects [50]. Generally, kaempferol and quercetin among the flavonones; delphinidin and cyanidin in the anthocyanins have emerged as powerful pest repellants along with potent antioxidant abilities [50]. Our study demonstrated a specific yet clear reconfiguration of the metabolites as part of the successful antibiosis and antixenosis response by utilizing cyanidin and delphinidin to produce more epicatechin-3-gallate and epigallocatechin. The high concentration of these flavonoids in infested plants, as compared to controls, suggested their induced accumulation in response to *H. armigera*. Why the plant is overproducing these epicatechins is an exciting question to answer.

## 8. Assessment of the Copy Number of Flavonoid Biosynthesis Genes in *C. platycarpus*

**Figure 4:**
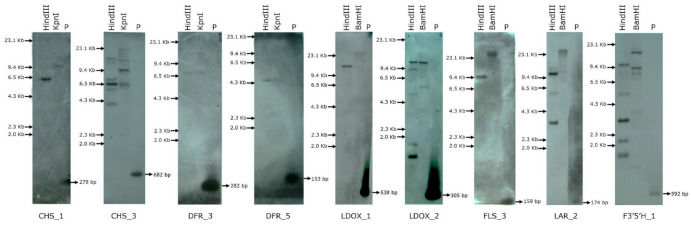
Gene copy number assessment of selected flavonoid pathway genes in *C. platycarpus*. Genomic Southern analysis of 9 genes digested with *HindIII*, *KpnI,* and *BamHI* and probed with DIG-labelled gene-specific probes; P: positive control.

There is a consensus in molecular biology that there is a relationship between copy number and expression of genes. It is expected that higher the gene copy number, higher could be the expression. In this study, we envisaged assessment of the number of copies of the isoforms of the selected flavonoid pathway genes that were upregulated during herbivory as we wanted to know whether the increased gene expression corroborated with their gene copy number. Based on the metabolite accumulation and dynamic gene expression, 9 genes- 2 isoforms of CHS, DFR, and LDOX and one isoform each of FLS, LAR and F3′5′H were selected for gene copy number analyses in *C. platycarpus* (Figure 4). It was observed that the genes coding for critical players in the flavonoid pathway were present in multiple copies. The results obtained from the study revealed that CHS_1, DFR_3, DFR_5, FLS_3, and LDOX_1 genes possessed a single copy, whereas CHS_3, F3′5′H_1, LAR_2, LDOX_2 genes were present in multiple copies. Besides this, being gatekeepers, CHS_3 and F3′5′H_1 genes showed higher expression (Figure 3) and the presence of multiple copies supported their role in defending herbivory. LAR_2 and LDOX_2 were also present in multiple copies because the expression of these genes was higher, and they are widely distributed among flavonoid pathways to produce different metabolites (Figure 4). These results authenticated a highly structured metabolic reprogramming to be happening in the wild relative to deter the herbivore.

## 9. Validation of Differentially Produced Flavonoids on the Growth and Development of *H. armigera*

*H. armigera,* has a highly evolving detoxification system prevalent that allows it to develop resistance against insecticides. Moreover, this herbivore being a polyphagous/generalist pest is exposed to a variety of chemicals from different hosts. Hence, antibiosis/antixenosis to *H. armigera* existing in the wild relatives is worth exploring so that it can be extrapolated to the cultivated germplasm. Studies emanating from the literature have demonstrated the presence of an active chemical barrier and identified the major players in wild chickpea [51] as well as wild pigeonpea [32]. Additionally, several secondary metabolites were independently tested on the herbivore to assess the antibiosis mechanism. Studies have reported that flavonoids such as chlorogenic acid, caffeic acid, quercetin, and protocatechuic acid were seen to be toxic to *H. armigera* larvae [52,53,54]. Some evidence for insect control also exists with kaempferol and naringenin [55,56]. Since there have been reports that have divulged the antibiosis role of flavonols such as kaempferol, rutin, and quercetin, [57] in the present study, we assessed the effect of anthocyanins and flavanols considering their increased accumulation in the wild relative. There has been no study thus far depicting such a validation on *H. armigera.*

**Figure 5:**
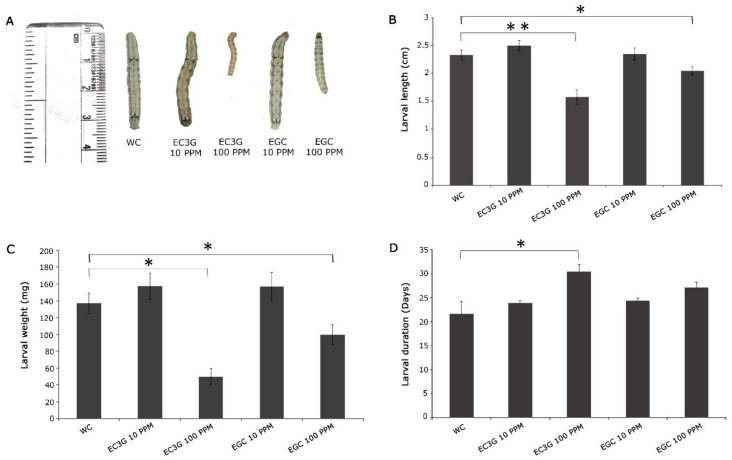
Diet overlay assay for the validation of selected flavonoids on *H. armigera.* Response of *H. armigera* larvae to artificial diet feeding assay incorporated with water-soluble flavonoids in 10 and 100 ppm concentrations. (**A**) Representative image of larvae that fed on flavonoids-incorporated artificial diet (**B**) Average length of larvae in cm; mean ± SE, *n* = 10 (**C**) Average larval weight in mg; mean ± SE, *n* = 10 (**D**) Average larval period in days; mean ± SE, *n* = 10. The larval length, weight, and duration were compared between control and respective treatments by student’s *t*-test; * *p* < 0.05; ** *p* < 0.001. WC: Water control; EC3G: Epicatechin-3-gallate; EGC: Epigallocatechin.

**Figure 6:**
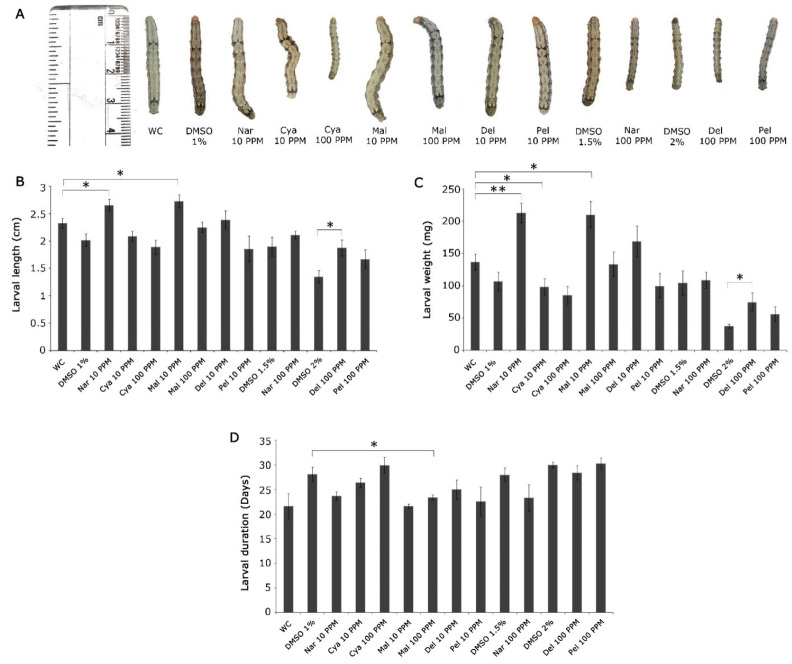
Performance of *H. armigera* larvae in artificial diet feeding assay incorporated with DMSO-soluble flavonoids (dissolved in WC, 1% DMSO, 1.5% DMSO, and 2% DMSO) in 10 and 100 ppm concentrations. (**A**) Representative image of larvae that fed on the selected flavonoids -incorporated artificial diet along with their respective controls (**B**) Average length of larvae in cm; mean ± SE, *n* = 10 (**C**) Average larval weight in mg; mean ± SE, *n* = 10 (**D**) Average larval period in days; mean ± SE, *n* = 10. The larval length, weight, and duration are compared between controls and respective treatments by student’s *t*-test; * *p* < 0.05; ** *p* < 0.001. WC: Water control; DMSO: Dimethylsulfoxide; Nar: Naringenin; Cya: Cyanidin; Mal: Malvidin; Del: Delphinidin; Pel: Pelargonidin.

The analyses focused mainly on the effect of two different concentrations of the selected flavonoids (10 ppm and 100 ppm) on larval growth and larval period. Since the selected flavonoids were both soluble in water as well as DMSO, the respective solvents were used as controls to compare their effects. It was observed that 10 ppm concentration did not have any negative effect on the feeding larvae of *H. armigera,* albeit the compounds and solvents (Figure 5 and Figure 6). However, drastic effects on the larval parameters were observed as the concentration of flavonoids increased to 100 ppm. The larval lengths across the metabolites varied between 1.58 cm (epicatechin-3-gallate -100 ppm) and 2.25 cm in malvidin (100 ppm). Further, the larval weight was lowest in those treated with 100 ppm epicatechin-3-gallate (49.8 mg; *p* < 0.05), while the larvae that fed on the diet incorporated with 100 ppm malvidin were the heaviest (133.4 mg). Nonetheless, the effect on larval length and weight was also observed in the larvae that fed on 100 ppm cyanidin, 100 ppm Naringenin and 100 ppm epigallocatechin depicting their role in the resistance response. A captivating observation made in the study was that feeding of the larvae on anthocyanins and flavan-3-ols resulted in a prolonged larval stage. The major contributors to this were epicatechin-3-gallate, epigallocatechin, and cyanidin 100 ppm. It was observed that the larval stage was delayed to an extent of 7.1 days (epicatechin-3-gallate) following the consumption of the metabolite-incorporated diet (Figure 5D).

Hence, it was clear that the wild relative methodically produced those metabolites in higher amounts that acted as anti-feedants as well as interfered in growth and development. In the present study, we categorically selected those metabolites for validation that accumulated in a dynamic manner during herbivory. However, we assessed the effect of malvidin which was present at a basal level in the plants through the herbivore attack (Figure 2). It was seen by the diet assay that both lower and higher amounts of malvidin seemed to benefit the larval growth and development and hence the wild relative chose to not increase its accumulation.

There have been varying reports on the putative role of anthocyanins in herbivore protection [58,59]. Additionally, studies on the effect of cyanidin, delphinidin, and cyanidin-3-glucoside from cotton demonstrated a reduction in the growth of tobacco budworm [60,61]. Further, petunia anthocyanins such as malvidin 3-*cistrans*-*p*-coumaroyl-rutinoside-5-glucoside resulted in growth retardation of corn earworm and cabbage looper [62]. This reinstates the novelty of the present study in deciphering the metabolites involved in the resistance response in the pigeonpea wild relative.

Plants respond to herbivory through strategic yet complicated mechanisms involving signal perception and transduction leading to transcriptional and metabolic reconfiguration. Further, it is also known that crop domestication resulted in the modification of secondary metabolites, thereby resulting in the loss of their ability to resist herbivores as compared to their wild counterparts. The present study comprehensively demonstrates the meticulous metabolic reprogramming occurring in the wild relative, *C. platycarpus,* and the combination of flavonoids portrayed in the antibiosis response to the attacking *H. armigera*. However, further investigations into the *cis* elements of these genes in the wild relative as well as their upstream regulators would divulge more fascinating cues that could be effectively translated for pod borer resistance. The emanating information and further in-depth characterization could henceforth pave the way for restoring the ability to manage this notorious pest in the cultivated *C. cajan.*

## Supplementary Materials

The following supporting information can be downloaded at: https://www.mdpi.com/article/10.3390/toxins14070455/s1, Table S1: List of primers used for qRT-PCR analyses; Table S2: List of primers used in probe designing for Southern blotting.
