# Supplementary material for: Deciphering of Pod Borer [Helicoverpa armigera (Hübner)] Resistance in Cajanus platycarpus (Benth.) Offers Novel Insights on the Reprogramming and Role of Flavonoid Biosynthesis Pathway
Source: Toxins (Basel). 2022 Jul 2;14(7):455. doi: 10.3390/toxins14070455 (PMC9325000; doi:10.3390/toxins14070455)
Supplement: Supplementary file 1 [file toxins-14-00455-s001.zip › toxins-1738244-supplementary.pdf]

toxins

MDPI

Article

Deciphering of Pod Borer [*Helicoverpa armigera* (Hübner)] Resistance in *Cajanus platycarpus* (Benth.) Offers Novel Insights on the Reprogramming and Role of Flavonoid Biosynthesis Pathway

Shaily Tyagi [1,2], Maniraj Rathinam [1], Pathour Rajendra Shashank [3], Nidhee Chaudhary [2], Ajit Kumar Shasany [1] and Rohini Sreevathsa [1,*]

[1] ICAR-National Institute for Plant Biotechnology, Pusa Campus, New Delhi 110012, India; shailys2709@gmail.com (S.T.); rmani607@gmail.com (M.R.); akshasany@gmail.com (A.K.S.)
[2] Centre for Biotechnology and Biochemical Engineering, Amity Institute of Biotechnology, Amity University, Noida 201313, India; nchaudhary@amity.edu
[3] Division of Entomology, ICAR-Indian Agricultural Research Institute, Pusa Campus, New Delhi 110012, India; spathour@gmail.com
* Correspondence: rohini.sreevathsa@icar.gov.in

Table S1. List of primers used for q-RT PCR analyses.

S.No.	Primer ID	Primer sequence (5'-3')	Amplicon size (bp)	Tm (ºC)
1	CHI_1FP	CGGCAGTGGCTGTTTATTTG	105	60
2	CHI_1RP	CTGTAGAAGTCAAGGGATTCCAG		
3	CHI_2FP	TCACCTACCCATCTGCATTTC	113	60
4	CHI_2RP	GCTGGTTCCTCCACGTATTC		
5	CHI_3FP	GGACTCCGTCCAATTCTTCAG	109	60
6	CHI_3RP	AGACACTTTCTCCGCGTATTG		
7	CHI_4FP	CCTCCGCCAAGACCTATTTC	116	60
8	CHI_4RP	TGATTGGATGGCTTGATCCTC		
9	CHS_1FP	AGCCTGTTCTGAGCCATAAG	97	60
10	CHS_1RP	CACATGCAAGAAGGATACAAAGG		
11	CHS_2FP	GAAGCTTGGTGAGCTGGTAATC	113	60
12	CHS_2RP	GACGAAGGCGATAAAGGAATGG		
13	CHS_3FP	CCTTGTTGTTCTCAGCCAAATC	128	60
14	CHS_3RP	TGACTACCAACTCACCAAACTC		
15	DFR_1FP	CCACTAGATCGATGCCACTTTC	102	60
16	DFR_1RP	TAGAGTACTGCAAACGCTACAAC		
17	DFR_2FP	ACATTGAGAGTTCCCTTCACAG	104	60
18	DFR_2RP	CAAGGTTGTCATGCTGTCTTTC		
19	DFR_3FP	CTCCTTCATCTTGACTCCGTTAG	127	60
20	DFR_3RP	TCACGTTCTTCGCTCCATTC		
21	DFR_4FP	AACGGTGAAGCGTCTGATATAC	144	60
22	DFR_4RP	CGAGAGAATCGATAAGCGAAGG		
23	DFR_5FP	TAGGCGAAAGAGCAGTGATAAG	104	60
24	DFR_5RP	CAAAGAACATGGCTTGGACTTC		
25	DFR_6FP	CCTATTCACAGGTTCCCAGAAG	129	60
26	DFR_6RP	GCTCTCTGCTGCTTCTCTTAC		
27	DFR_7FP	GGCAGTTTCAGCCAATCTTTC	143	60
28	DFR_7RP	AAGGAGCATCCACCCATAAAG		
29	F3′5′H_1FP	ACAGCAGGAACAGACACATC	109	60
30	F3′5′H_1RP	CTATCACTGCGTCCATCTCTTC		
31	FLS_1FP	ACGTTAACAGCATCCCTTCTC	107	60

32	FLS_1RP	CACAAGGTGCCTTTGGATAAAC		
33	FLS_2FP	TCTTCGTTGTGAACCTGTATCC	123	60
34	FLS_2RP	TAACCTGAAGCCCTCCAATTC		
35	FLS_3FP	CAACCAACAATGCTTGGAGAG	100	60
36	FLS_3RP	GAGCTCCAGCTCCATATAATCC		
37	LAR_1FP	GAGGGCTACAGAAGGAAGATTG	112	60
38	LAR_1RP	GGTAAATGCAGAGGTGTGAAAG		
39	LAR_2FP	ACTGGTCTGGTTATGGATGAAAG	113	60
40	LAR_2RP	CATGCTTCCTTCTCTGCTAGTG		
41	LAR_3FP	ACGTGGTGATTTCTCTTGTAGG	109	60
42	LAR_3RP	CCAAACTCTGAAGGCACAAAC		
43	LDOX_1FP	CGAGTATGCAAAGAGACTAAGGG	107	60
44	LDOX_1RP	CCATTCCTCCAACTTCCTTCTC		
45	LDOX_2FP	CATGCACCAAGCTCACTTTC	115	60
46	LDOX_2RP	GTGAAGGAGTGGTTAAGCTAGG		
47	UFGT_1FP	GGTAGCGGTGTTCTCTTCTATC	122	60
48	UFGT_1RP	CCAAACTGCTCTTCAAGGTAATC		
49	UFGT_2FP	GCAAGGATGAGAAGTGGGATAG	104	60
50	UFGT_2RP	TTCATCACTTAGTGTCACCTCAC		
51	UFGT_3FP	GAGCTAGCGCTCAAGTCTATTG	113	60
52	UFGT_3RP	ACAACTGGGTGTGGATAAGATG		
53	UFGT_4FP	GGGTCATAACACTGATGTCTTCC	105	60
54	UFGT_4RP	TTCACTTGATCCTCACCCAAAG		
55	UFGT_5FP	GACCGGAACGATAGGCAATAC	113	60
56	UFGT_5RP	GCTTCCAGCGTTTCATGTTG		

Table S2. List of primers used in probe designing for Southern blotting.

S.No.	Primer ID	Primer sequence (5'-3')	Amplicon size (bp)	Tm (ºC)
1	PRB_CHS1_FP	GGGATAGTCATAACAGCGTTCTC	278	60
2	PRB_CHS1_RP	CCACCGATTAAACGGCTCTG		
3	PRB_CHS3_FP	AGCAGCAAAGGCTATAAAGGAG	682	60
4	PRB_CHS3_RP	GTTACCATACTCACTGAGCACTTC		
5	PRB_DFR3_FP	TATTGCTATGGGAAAGCTGTGG	282	60
6	PRB_DFR3_RP	GGAGGATTCAGCACACAAGTATC		
7	PRB_DFR5_FP	GATATTCCAGAAGAATTGGAGCTTG	153	60
8	PRB_DFR5_RP	TGGAGCTTCTGCAGGTTTAG		
9	PRB_F3'5'H1_FP	CAGACACATCCTCGAGCATAATAG	592	60
10	PRB_F3'5'H1_RP	GTCAACCTAGGGCTAACGAAAG		
11	PRB_FLS3_FP	CTACGTCGTCAATCATGGGATAAG	159	60
12	PRB_FLS3_RP	TCAGGATCAAGTAACTCATCAAGAAC		
13	PRB_LAR2_FP	CTCAGGAGTATTGAATGTGTTGAAAG	174	60
14	PRB_LAR2_RP	GTGGGTGGCTTTGCATTTG		
15	PRB_LDOX1_FP	GTTACAAGCGAGTATGCAAAGAG	538	60
16	PRB_LDOX1_RP	CGTCCTTTCTGAAGAGTTTGTG		
17	PRB_LDOX2_FP	GTGAAGGAGTGGTTAAGCTAGG	305	60
18	PRB_LDOX2_RP	GTCACCCATGTTGATGATGAAAG		
